# Delayed Radial Nerve Palsy after Closed Reduction of a Pediatric Humeral Shaft Fracture

**DOI:** 10.1155/2017/9723497

**Published:** 2017-12-28

**Authors:** Robert Runner, Emily Whicker, Sayan De

**Affiliations:** ^1^Department of Orthopaedics, Emory University, 59 Executive Park South, Suite 3000, Atlanta, GA 30329, USA; ^2^Pediatric Orthopaedic Associates, 6 Executive Park Drive, Suite 10, Atlanta, GA 30329, USA

## Abstract

Humeral shaft fractures are common in the United States and may be associated with radial nerve injuries due to their close anatomic relationship in the spiral groove. Most radial nerve palsies are found at presentation due to the initial trauma; however, they can present secondary to orthopaedic intervention following reduction. In this case report, we present a case of delayed radial nerve palsy in a pediatric patient that was identified four days after closed reduction and splinting which required open reduction, nerve exploration, and internal fixation. Fortunately, full motor and sensory recovery was observed at 6 weeks post-op. A unique aspect of this case is that immediate postreduction exam in the emergency department showed no signs of injury or entrapment of the radial nerve.

## 1. Introduction

Humeral shaft fractures are common in the United States, with more than 237,000 cases occurring each year [1]. Fractures of the humerus occur in a bimodal distribution with the majority occurring in males under the age of 25 and in women over the age of 50 [1]. One known association of humeral fractures is the radial nerve palsy. Treatment of the radial nerve palsy can be conducted surgically or nonsurgically. Indications for operative treatment include an open injury or a closed injury with a high-energy mechanism of injury in conjunction with a suspicion of radial nerve laceration. In such cases, early exploration of the fracture site is recommended in order to provide ample opportunity for the nerve to obtain functional recovery [2]. In the pediatric population, a vast majority of humeral shaft fractures are treated nonoperatively [3].

Due to the anatomic relationship of the radial nerve with the humeral shaft at the lateral supracondylar ridge, the radial nerve palsy is the most common nerve injury following humeral shaft fractures [1]. At this location, the radial nerve is tethered as it pierces the intermuscular septum and cannot accommodate a lateral displacement of the distal humeral fragment [4]. Radial nerve palsies can be classified as partial or complete and further classified into primary, secondary, or delayed. Secondary radial nerve palsies occur during treatment of the fracture and account for 20% of all nerve palsies [1]. Primary radial nerve palsies resolve fully in 87.3% of patients, though it has been suggested that more aggressive treatment options with early exploration of the injury could improve time to functional recovery [1].

We report a case of delayed radial nerve palsy occurring four days following closed reduction and splinting of a midshaft humeral fracture in a pediatric patient.

## 2. Case Report

**Figure 1:**
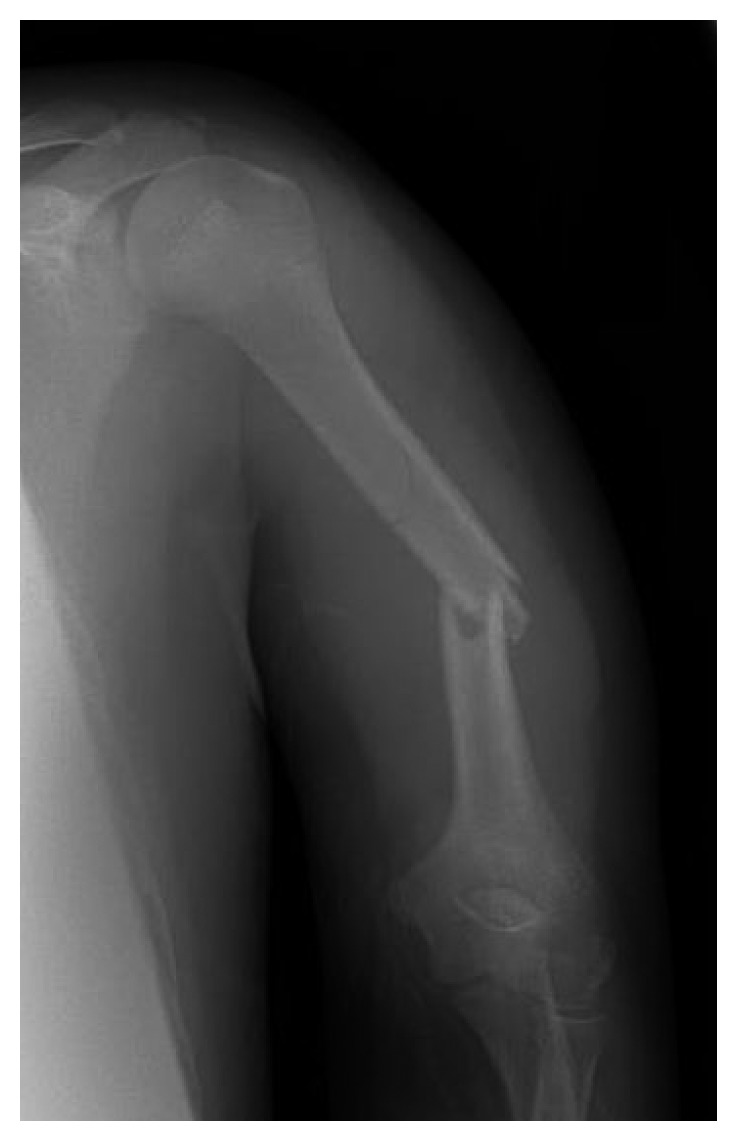
Anteroposterior radiograph showing a displaced midshaft humerus fracture in 35° varus angulation.

**Figure 2:**
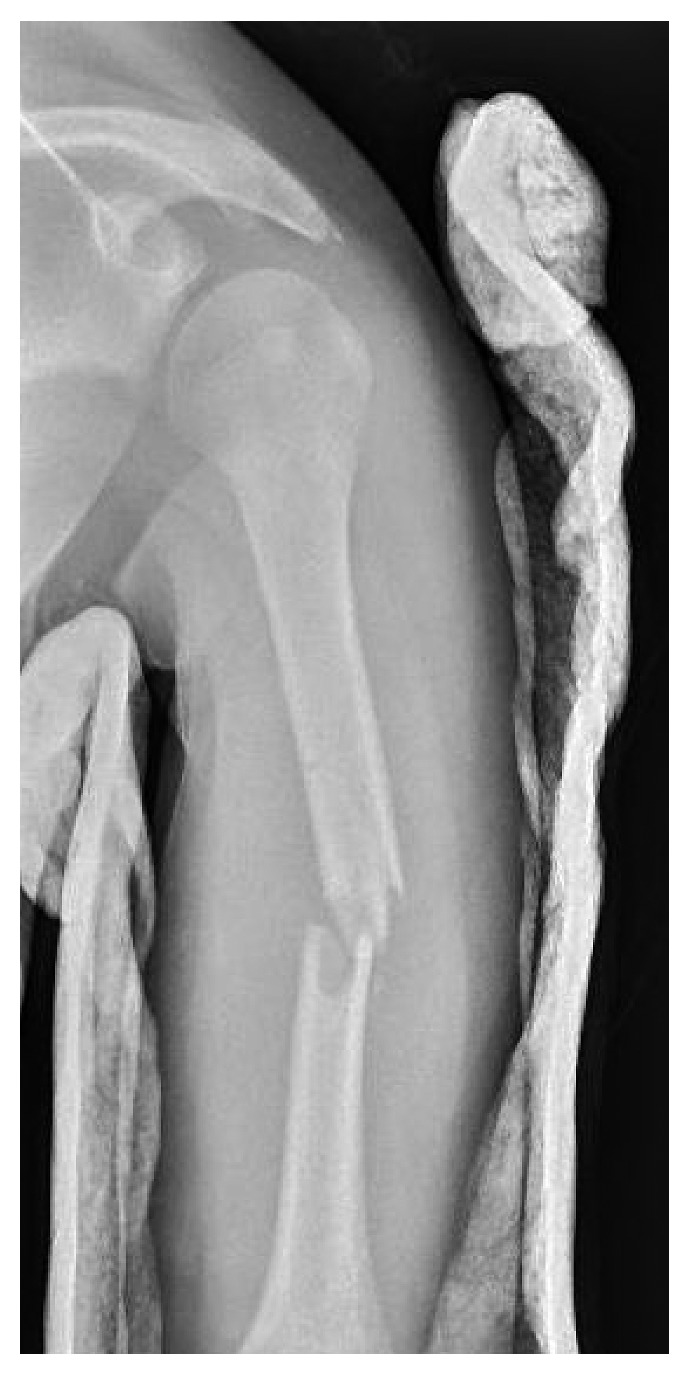
Anteroposterior radiograph following reduction and coaptation splinting showing some mild distraction but overall improvement of alignment of the humerus fracture with acceptable residual 15° varus angulation.

JC is a 12-year-old male who was involved in a T-bone motor vehicle collision one day prior to presentation at our Level 1 Pediatric Trauma Center. He was initially treated at an outside hospital for a closed left humeral shaft fracture, and a coaptation splint was placed with no reduction; radial nerve function was reported intact. The patient had continued pain in the arm and was brought to our emergency department (ED) by his father for further evaluation. Upon evaluation in the ED, this was an isolated closed injury, and the patient was fully neurovascular intact in sensation with full motor strength in all peripheral nerves. Initial radiographs (Figure 1) showed a displaced midshaft humerus fracture of 35° varus angulation. After discussion with the patient's father, given his continued pain and significant angulation, a closed reduction under conscious sedation was performed, and a coaptation splint was placed. Postreduction radiographs (Figure 2) showed some mild distraction but with overall acceptable alignment in 15° varus angulation. After the patient was fully awake from sedation, a thorough neurovascular exam was obtained (see Supplemental Video 1) which revealed intact sensation and full strength in the median, radial, ulnar, posterior interosseous, and anterior interosseous nerves with a strong 2+ radial pulse. There was no pain with passive range of motion, and his compartments were soft and compressible. The patient and family were counseled on signs and symptoms of compartment syndrome as well as on signs of the radial nerve palsy to return to the ED with any concern. They planned to follow up with a pediatric orthopaedist within 1 week.

**Figure 3:**
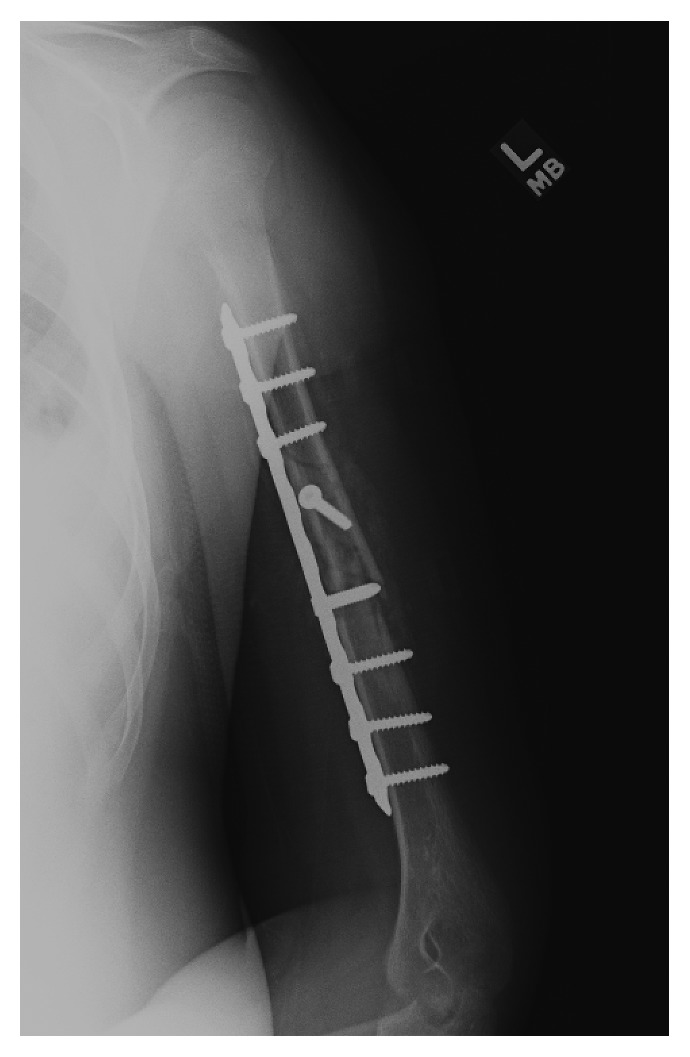
Anteroposterior radiograph following open reduction internal fixation with intact hardware and the fracture in anatomic alignment.

Four days post reduction, the patient returned to our office for clinical evaluation. On exam, he had a dense motor and sensory radial nerve palsy (Supplemental Video 1) with 0/5 strength in the extensors of his thumb, fingers, or wrist. He had complete anesthesia in radial nerve distribution. Compartments remained soft with intact radial pulse and no signs of other trauma. His radiographs showed no change in position. Discussion was had with the patient and father regarding observation versus exploration of the nerve and surgical fixation. They elected for exploration, and he was scheduled for surgery. Repeat evaluation on day of surgery, 11 days post injury, showed continued dense radial nerve palsy. The patient underwent open reduction internal fixation via an anterolateral approach using a 9-hole LC-DC plate (Figure 3). The radial nerve was explored and was found to be draped and stretched over a sharp fracture fragment. There was significant edema and ecchymosis around the perineural tissue; however, the radial nerve was noted to be contused but in continuity. The patient was made non-weight-bearing with range of motion as tolerated and was admitted overnight for observation and pain control but showed no change in nerve function immediately post-op.

The patient continued to follow up at routine intervals. His radial nerve palsy showed no improvement at 2 weeks post-op. At his next clinic visit at 6 weeks post-op, the patient had full recovery of his motor and sensory radial nerve function (Supplemental Video 1). He was seen again at 3 months post-op with full shoulder/elbow/wrist/finger range of motion and 5/5 strength in all muscle groups. His fracture was fully healed on imaging, and he progressed with no residual functional deficits.

## 3. Discussion

Previous case reports have shown delayed radial nerve palsy following conservative management of humerus fractures [5]. However, our case was distinct from the traditional Holstein–Lewis humeral shaft fracture in that our pediatric patient sustained a displaced midshaft humerus fracture. The Holstein–Lewis fracture pattern is classically described as a spiral distal third humeral shaft fracture and is traditionally associated with radial nerve palsy as the nerve can become lacerated or entrapped by the displaced fragments due to the limited mobility of the nerve at the distal end of the humerus [6]. Further studies have shown that the radial nerve is at risk in other humeral shaft fracture patterns, even midshaft fractures [4].

Radial nerve palsies occur in 2–15% of humeral shaft fractures [1]. Patients who demonstrate a primary radial nerve palsy have a reported spontaneous recovery rate of over 70% [7]. In the management of nerve palsy, nerve conduction study with electromyography (EMG) studies could be obtained at three months post injury, as motor potential should be recovered by this point, and these nerve conduction data will enable the clinician to determine if the injury should require surgical intervention [8]. Overall, the most common cause of radial nerve palsy in closed fractures is neurapraxia, and it is likely that our delayed radial nerve palsy was due to nerve contusion/neurapraxia [9].

There is substantial evidence for the conservative treatment of radial nerve palsies that occur at the time of humeral shaft fractures due to the high rate of spontaneous recovery [2, 10]. Early surgical exploration of the nerve is generally indicated for cases in which there was an open fracture, a vascular injury, a high-velocity gunshot wound, or a severe soft tissue injury [1]. While our patient did not meet these criteria, given that our patient's radial nerve was intact at initial presentation and after immediate reduction, we were concerned for ongoing injury to the nerve. After discussion of conservative and operative treatment with the family, we opted for surgical exploration of the nerve. Our exploration revealed that the radial nerve was not wrapped or entrapped in the fracture, but rather, it was draped in tension over a large fracture spike. This close contact with the fracture and sharp edges of the bone likely contused the nerve, producing the clinically delayed palsy as the contusion developed and worsened over the next several days.

Additionally, this case highlights the necessity of a postreduction exam. Nerve function should always be assessed before and after reduction, with complete and thorough documentation in the medical record. Additionally, to ensure accuracy, it is imperative that any patient who requires conscious sedation for reduction must return to full consciousness and alertness before obtaining the postreduction exam. Even though our patient was fully awake during the exam (Supplemental Video 1), delayed palsies can still occur. Finally, it is important to counsel not only the pediatric patients but also their parents and families on the signs and symptoms of compartment syndrome and nerve damage, as they will be able to reassess the patient to detect red flag symptoms on a daily basis at home.

In conclusion, our case of delayed radial nerve palsy in a pediatric patient with the humeral shaft fracture highlights the importance of careful examination and thorough documentation of neurovascular status prereduction, immediate postreduction, and with subsequent clinical evaluation. Should delayed radial nerve palsy occur, we suggest surgical exploration with possible plating of the fracture. The risks of the surgery are relatively minimal as long as the surgeon is comfortable with the approach and potentially could improve the time to recover nerve function, due to the removal of the fracture fragment. A majority of radial nerve palsies will recover within several weeks, as our patient did, and patient education is paramount.

## Supplementary Materials

Supplementary Materials
*Video 1*. Video presenting the case. The intact postreduction neurovascular exam is shown, followed by the subsequent motor exam on postreduction day 4, demonstrating delayed radial nerve palsy. His clinical exam at six weeks is also included, revealing a full return of the patient's radial nerve function.
